# Integrated Role of Microbial, Fungal, and Plant-Derived Interventions in the Management of Celiac Disease: A Narrative Review

**DOI:** 10.7759/cureus.102471

**Published:** 2026-01-28

**Authors:** Karolina Kubala, Tomasz Pietrucha, Mikolaj Goldyn, Magdalena Grabinska, Paulina Halik, Justyna Jusiak

**Affiliations:** 1 General Practice, Provincial Specialist Hospital Named After J. Gromkowski, Wroclaw, POL; 2 General Practice, Wrocław Medical University, Wroclaw, POL; 3 General Practice, Beskid Oncology Center – Municipal Hospital of John Paul II, Bielsko-Biala, POL; 4 General Practice, Józef Struś Multispecialty Municipal Hospital With the SPZOZ (Samodzielny Publiczny Zakład Opieki Zdrowotnej) Care and Treatment Facility, Poznan, POL; 5 General Practice, The Baptism of Poland Memorial Hospital, Gniezno, POL; 6 General Practice, Hospital of the Monument of the Baptism of Poland, Gniezno, POL

**Keywords:** autoimmune enteropathy, celiac disease, cysteine proteases, enzymatic therapy, gluten-free diet, larazotide acetate, microbiota modulation, probiotics, prolyl endopeptidases

## Abstract

Celiac disease (CeD) is a chronic autoimmune enteropathy triggered by gluten ingestion in genetically susceptible individuals carrying human leukocyte antigen (HLA)-DQ2 or HLA-DQ8 haplotypes. While a strict gluten-free diet (GFD) remains the primary treatment, many patients experience persistent symptoms and incomplete mucosal recovery, often due to accidental exposure. This narrative review evaluates complementary biological strategies that enhance gluten management beyond dietary avoidance. We discuss enzymatic approaches using bacterial and fungal prolyl endopeptidases (PEPs) and engineered enzyme combinations, such as latiglutenase, to degrade immunogenic peptides in the gastrointestinal tract. Furthermore, we examine the restoration of intestinal barrier integrity through zonulin antagonists such as larazotide acetate. The role of gut microbiota modulation with probiotics, such as Lactobacillus and Bifidobacterium strains, is analyzed for its potential to reduce inflammation and support gliadin degradation. Additionally, plant-derived cysteine proteases from sprouting cereals are presented as promising agents for gluten detoxification. Finally, the application of enzymatic degradation in food processing is considered to improve the safety and affordability of gluten-free products. Together, these strategies offer a multidimensional framework for enhancing clinical outcomes and quality of life for individuals with CeD.

## Introduction and background

Celiac disease (CeD) is a chronic autoimmune disorder characterized by an inappropriate immune response to dietary gluten, affecting approximately 1-2% of the global population, with a higher prevalence in females [1]. The prevalence varies by region, with higher rates in Europe and North America and lower rates reported in East Asia, Sub-Saharan Africa, and South America. Global incidence rates vary from 17.4 per 100,000 person-years in women and 7.8 in men. Both prevalence and incidence have increased over the past three decades due to improved detection and changes in dietary habits, including greater consumption of convenience foods and gluten [2]. CeD develops in genetically susceptible individuals, most commonly those carrying human leukocyte antigen (HLA)-DQ2 or HLA-DQ8 haplotypes [3]. The disorder is triggered by the ingestion of gluten, a composite of storage proteins in wheat, barley, and rye, which are rich in proline and glutamine residues. These proteins are highly resistant to complete gastrointestinal digestion, which contributes to the persistence of immunogenic peptides in the gut [4].

Clinically, CeD presents a broad spectrum of manifestations. Classic gastrointestinal symptoms include chronic diarrhea, abdominal pain, bloating, and malabsorption, while extraintestinal features can include iron-deficiency anemia, osteoporosis, dermatitis herpetiformis, neurological disorders, and infertility [5]. Studies indicate that a significant proportion of patients, up to 30-40% of adults, do not achieve complete mucosal recovery despite strict adherence to a gluten-free diet (GFD) [6]. Persistent symptoms are reported by 40.7-42.2% of patients, including fatigue, abdominal discomfort, and other non-gastrointestinal manifestations, highlighting the limitations of current dietary management [7].

The standard treatment for CeD, the GFD, involves complete avoidance of gluten-containing grains and products, including wheat, barley, rye, and their derivatives. While effective at reducing symptoms and promoting mucosal healing, the diet is highly restrictive, socially challenging, and often associated with financial burden. Given these challenges, research has increasingly focused on complementary therapeutic strategies that go beyond gluten avoidance. These include enzymatic degradation of immunogenic gluten peptides, restoration of intestinal barrier integrity, and modulation of gut microbiota to reduce inflammation and improve tolerance. Understanding the immunopathogenesis of CeD is critical to developing these novel interventions. This narrative review explores how interventions sourced from the microbial, fungal, and plant kingdoms can help manage CeD, focusing on mechanisms for gluten detoxification that the human body cannot perform on its own.

## Review

Pathophysiology of celiac disease and therapeutic targets

The pathogenesis of CeD is initiated when gluten proteins from wheat, barley, and rye, rich in proline and glutamine, resist complete gastrointestinal digestion. Due to this process, immunogenic peptides are produced, including the highly stimulatory 33-mer α-gliadin and 26-mer 𝛾-gliadin sequences. These products are particularly potent T-cell activators and drive the initial immune cascade [8]. These peptides cross a compromised intestinal epithelial barrier, facilitated by increased permeability, and reach the lamina propria, where tissue transglutaminase (tTG) deamidates glutamine residues. Furthermore, negatively charged peptides are generated, which have enhanced affinity for HLA-DQ2 and HLA-DQ8 molecules on antigen-presenting cells [8]. Presentation of these complexes activates gluten-specific CD4+ T cells, triggering a Th1-type pro-inflammatory response with cytokines such as interferon-gamma (IFN-𝛾) and interleukin-21 (IL-21) [9].

Simultaneously, innate immune mechanisms, particularly interleukin-15 (IL-15) signaling, promote expansion and cytotoxic activity of intraepithelial lymphocytes (IELs) and induce expression of major histocompatibility complex class I-related chain A (MICA) ligands and natural killer group 2D (NKG2D) receptors on enterocytes, resulting in direct epithelial destruction and villous atrophy [9]. Anti-inflammatory mediators, such as interleukin-10 (IL-10) and transforming growth factor-beta (TGF-beta), are upregulated but are insufficient to counteract the autoimmune response; they contribute to IgA production and tissue repair but do not prevent ongoing mucosal injury [10].

The genetic predisposition, primarily HLA-DQ2 (present in ~95% of patients) and HLA-DQ8 (~5% of patients), is necessary for disease development, although additional unknown genetic factors likely contribute. Homozygosity for HLA-DQB1*02 is associated with higher disease risk, and first-degree relatives and monozygotic twins show increased susceptibility [8]. The combined adaptive and innate immune responses produce hallmark histological changes. Conditions such as villous atrophy, crypt hyperplasia, and dense IEL infiltration can cause malabsorption. Even minimal gluten exposure, including cross-contamination, can sustain immune activation and prevent mucosal healing. Persistent immune activation in severe or refractory cases may lead to refractory celiac disease (RCD) or enteropathy-associated T-cell lymphoma (EATL) [11].

Challenges of a gluten-free diet

Although a strict GFD is the only effective treatment for CeD, adherence is difficult for many patients because gluten is ubiquitous in processed foods and cross-contamination risks are widespread in restaurants and shared kitchens [12]. Many individuals with CeD report that gluten-free products are expensive and difficult to find, which limits dietary choices and creates a financial burden that affects everyday living. Studies report that gluten-free products are, on average, two to four times more expensive than their gluten-containing counterparts [13]. Limited availability of affordable gluten-free options contributes to barriers to adherence, particularly in regions with fewer specialty products, and these barriers can compound over time [12].

Adherence challenges have important implications for mental health. Individuals with CeD frequently experience psychological distress, including anxiety about accidental gluten exposure. Economic strain from purchasing costly gluten-free foods is associated with lower scores in emotional well-being [12]. Moreover, social and emotional fears related to the diet, such as embarrassment and worry about the availability of safe foods, are significant contributors to reduced quality of life, especially in women with CeD [14]. The psychosocial struggle of maintaining a GFD can include social limitations, such as difficulty participating in family meals or travel. Adults living with CeD also report persistent changes in mood and shifts in self-perception regarding the diagnosis [15]. Despite strict adherence, many patients continue to report ongoing health issues, such as fatigue and abdominal discomfort [16].

Enzymatic gluten degradation: prolyl endopeptidases (PEPs)

Prolyl endopeptidases (PEPs) are enzymes that specifically cleave proline-rich regions of gluten. Those regions contain multiple T-cell-immunogenic epitopes responsible for triggering the immune response in individuals with CeD. The best-characterized bacterial PEPs are derived from Flavobacterium meningosepticum (FM-PEP), Sphingomonas capsulata (SC-PEP, also known as ALV002), and Myxococcus xanthus (MX-PEP) [17]. The physicochemical and biochemical properties of FM-PEP and SC-PEP have been extensively examined, and their therapeutic potential in CeD has been thoroughly explored [18].

A comparative study assessed the gluten-degrading activity of FM-PEP, SC-PEP, and MX-PEP using two substrates: the strongly immunogenic 33-mer peptide and a shorter, related α-gliadin sequence. Although gliadins contain many T-cell-stimulatory peptides, two sequences, 33-mer α-gliadin and 26-mer 𝛾-gliadin, are considered particularly potent T-cell activators in the initiation and progression of CeD [19]. PEPs preferentially cleave proline-glutamine (P-Q) bonds, which are abundant in immunogenic gluten peptides and resistant to mammalian proteases. Despite their shared substrate preference, the three enzymes differ in specificity: SC-PEP and MX-PEP show stronger activity against the shorter peptide PQPQLPYPQPQLP, whereas FM-PEP more effectively cleaves the full-length 33-mer. SC-PEP, however, displays limited activity toward the 33-mer. All three PEPs remain structurally stable and enzymatically active at pH 6-7, corresponding to the mildly acidic environment of the upper small intestine where CeD pathology develops. These enzymes also demonstrate moderate resistance to pancreatic proteases and acidic conditions but are fully inactivated by pepsin, indicating the need for protection from gastric degradation. In vivo studies show that these PEPs retain proteolytic activity within the small intestine of rats, suggesting resistance to brush border membrane peptidases. This supports the potential use of bacterial PEPs as therapeutic agents capable of degrading immunogenic gluten peptides within the intestinal lumen [20].

Aspergillus niger-derived prolyl endoprotease (AN-PEP)

Another promising enzyme is AN-PEP, a prolyl endoprotease produced by Aspergillus niger [21]. AN-PEP efficiently degrades intact gluten and eliminates the T-cell-stimulatory activity of gluten digests generated under simulated gastrointestinal conditions [22]. Unlike bacterial PEPs from Streptomyces griseus (SC), Fusarium (FM), or Mucor (MX), which function optimally at pH 7.0-8.0 and are therefore inactive in the stomach, AN-PEP remains active at lower pH values, with maximal activity at pH 4.0-5.0. This enables gluten degradation directly in the stomach, eliminating the need for enteric-coated formulations and potentially enhancing early gluten detoxification [18].

Studies have shown that AN-PEP can degrade small amounts of gluten when consumed as part of a complex meal, indicating its potential role in reducing the consequences of accidental gluten ingestion [23]. However, one clinical trial failed to meet its primary endpoint because the placebo group did not exhibit symptom worsening, limiting the ability to detect a treatment effect [24]. Another study found no significant differences between placebo and AN-PEP groups in gluten-immunogenic peptides (GIPs) or other biomarkers, noting that “AN-PEP should not be used as a substitute for a GFD but rather as a supplement to support digestion of occasional or inadvertent gluten consumption” [25]. Although the efficacy of orally administered AN-PEP has not yet been conclusively demonstrated, the enzyme shows potential for use in the development of gluten-reduced or gluten-detoxified food products, as it can sufficiently lower gluten immunoreactivity in certain food matrices [26].

Latiglutenase (ALV003)

Latiglutenase (IMGX003, formerly ALV003) is a combination of bacterial and plant-derived proteases engineered to break down immunogenic gluten peptides before they reach the small intestine. It contains equal proportions of ALV001, a recombinant cysteine endoprotease derived from barley (EP-B2), and ALV002, a recombinant SC-PEP. These peptides are notably resistant to gastrointestinal digestion and contribute to the T-cell-mediated immune response characteristic of CeD; therefore, enzymatic degradation serves as a rational approach for reducing peptide immunogenicity. Latiglutenase acts locally in the stomach during meals, lowering the availability of immunogenic peptides before they can initiate downstream autoimmune activity [27].

A double-blind, placebo-controlled phase 2 trial showed that a daily 1200-mg dose of latiglutenase reduced gluten-induced mucosal injury in patients challenged with 2 g of gluten daily for six weeks. The treatment lessened reductions in villus height-to-crypt depth ratio and limited increases in IEL counts, with trends toward decreased abdominal pain, bloating, and fatigue [28]. Real-world data further indicate that seropositive patients experiencing persistent symptoms despite a GFD exhibit dose-dependent improvements in symptom severity and quality of life when using latiglutenase [29]. However, findings across studies are not fully consistent. A separate meta-analysis reported that latiglutenase did not significantly improve adverse events or histological outcomes in patients with CeD [30]. Therefore, although current evidence suggests that latiglutenase may help reduce the clinical impact of inadvertent gluten exposure, further high-quality randomized controlled trials are required, and existing sources remain inconclusive [31].

Larazotide acetate

Larazotide acetate (LA), an 8-amino-acid peptide, aims to restore epithelial tight-junction integrity disrupted by zonulin, a physiological modulator that increases intestinal permeability and allows gluten peptides to trigger immune responses in CeD [32]. Orally administered as an adjunct therapy, LA acts as a zonulin antagonist, promotes tight junction protein rearrangement, and inhibits myosin light chain kinase. As a result, it collectively reduces intestinal permeability and supports barrier function in patients with gliadin-induced epithelial disruption [33].

An exploratory double-blind, placebo-controlled trial was performed where 184 CeD patients on a GFD received LA (1, 4, or 8 mg) or placebo while consuming 2.7 g of gluten daily for 6 weeks. The 1-mg dose reduced gluten-induced symptoms and limited rises in anti-transglutaminase IgA. Intestinal permeability, assessed by the lactulose-to-mannitol (LAMA) ratio, showed no significant differences between groups. LA was well-tolerated, suggesting symptom and immune benefits despite unchanged permeability [34]. In a multicenter, randomized, double-blind, placebo-controlled trial, adults with CeD on a stable GFD received LA (0.5, 1, or 2 mg) three times daily for 12 weeks. The 0.5-mg dose significantly reduced gastrointestinal symptoms compared with placebo and improved exploratory outcomes, such as abdominal pain, symptomatic days, and non-gastrointestinal symptoms like headache and fatigue. Higher doses (1 and 2 mg) showed no benefit. LA was well-tolerated, supporting its potential as a therapy for patients with persistent symptoms despite a GFD [35]. Unfortunately, the trial stopped at phase 3 in 2019 with no further follow-up [36].

Probiotics and plant-derived interventions

Patients with CeD often display a disrupted gut microbiota: studies consistently report a reduction in beneficial bacteria (e.g., Bifidobacterium and Lactobacillus) and an increase in potentially pathogenic or inflammatory species compared to healthy controls [30]. Even after years on a strict GFD, many patients do not fully restore a healthy microbial balance, which may contribute to ongoing symptoms despite mucosal healing [37]. Probiotics have been explored to enhance gut microbiota composition, reduce gluten immunogenicity, and improve intestinal barrier integrity. A recent in silico analysis explored the potential of Lactobacillus rhamnosus as a gluten-degrading probiotic. Complete genomes of 49 strains were annotated and screened for peptidases with domains associated with known gluten-digesting enzymes. Among the 61 identified peptidases, nine, including aminopeptidase N, neutral endopeptidase, oligoendopeptidase F, and proline-specific enzymes, showed structural similarity to enzymes capable of breaking down toxic gliadin peptides. These findings suggest that Lactobacillus rhamnosus may possess previously unrecognized gluten-degrading potential. With further functional validation, this widely used probiotic could contribute to the development of microbial and probiotic therapies for CeD [38].

Interestingly, another study shows that Bifidobacterium longum CECT 7347 can counteract several harmful effects of gliadin on the intestine. In animals fed gliadin, the probiotic reduced inflammation and normalized key protein pathways disrupted by gluten exposure, indicating a protective influence on gut function. Although its effect was less pronounced in animals with severe, IFN-𝛾-induced immune activation, Bifidobacterium longum still showed no detrimental impact on the intestinal proteome when administered alone. Overall, the findings highlight Bifidobacterium as a promising probiotic capable of mitigating gluten-induced mucosal damage [39]. More broadly, modulating the gut microbiota by restoring beneficial bacterial populations and reducing dysbiosis may offer a great addition to a GFD. This strategy can potentially improve control of the symptoms, reduce inflammation, and enhance mucosal resilience [40].

Moreover, enzymes derived from plants, especially cysteine proteases from sprouting cereals, offer a complementary and promising route for gluten detoxification. Plant-derived cysteine proteases, particularly from barley (Hordeum vulgare), have been demonstrated to degrade gliadin proteins from various wheat species during germination [41]. Within this context, different families of cysteine proteases, including C1A (papain-like) and C13 (legumains), exhibited variable efficiency, with specific barley enzymes, such as HvPap-4 and HvPap-6, achieving near-complete gliadin degradation after 12 hours of incubation, as confirmed by Western blot analyses [42]. Furthermore, studies on proteases from germinating cereals, including barley, wheat, and rye, have shown that these enzymes can hydrolyze toxic gliadin peptides into fragments shorter than nine amino acids, effectively reducing their immunogenic potential [43]. Finally, the mechanism of gluten degradation mediated by plant proteases during cereal germination is considered a promising strategy for lowering pro-inflammatory and immunogenic protein content, providing a potential complementary approach for managing CeD [44].

## Conclusions

CeD remains a complex autoimmune disorder in which strict adherence to a GFD is currently the only fully effective treatment. However, the limitations of the diet underscore the need for complementary therapeutic strategies. Research has increasingly highlighted the potential of other biological kingdoms, such as bacteria, fungi, and plants, in supporting the management of CeD. Bacterial and fungal enzymes demonstrate the capacity to degrade immunogenic gluten peptides resistant to human digestion. These enzymes, alone or in combination with plant-derived proteases, offer a promising approach to reducing gluten immunogenicity and mitigating the risk of immune activation from trace gluten exposure. Clinical investigations suggest potential benefits in symptom reduction, though further trials are required to establish their role as adjuncts. Modulation of the gut microbiota represents an additional complementary pathway, where probiotics show the potential to restore microbial balance and reduce inflammation. Beyond clinical applications, enzymatic gluten degradation can also be leveraged during food production to enhance the safety, quality, and affordability of gluten-free products. Taken together, these insights highlight a multifaceted strategy that could synergistically enhance the quality of life for individuals with CeD. While gluten avoidance remains essential, integrating these biologically derived approaches may improve symptom control and overall well-being of patients. It follows that the continuation of current research and the search for new therapeutic solutions in CeD are extremely necessary and indispensable.
